# Management of persistent acromegaly following primary therapy: The current landscape in the UK

**DOI:** 10.1002/edm2.158

**Published:** 2020-06-09

**Authors:** Nikolaos Kyriakakis, Khyatisha Seejore, Ahmed Hanafy, Robert D. Murray

**Affiliations:** ^1^ Department of Endocrinology Leeds Centre for Diabetes & Endocrinology Leeds Teaching Hospitals NHS Trust Leeds UK; ^2^ Leeds Institute of Cardiovascular and Metabolic Medicine University of Leeds Leeds UK

**Keywords:** acromegaly, cabergoline, pegvisomant, persistent disease, radiotherapy, somatostatin analogues

## Abstract

Acromegaly is the clinical consequence of chronic exposure of the tissues to excess GH and IGF‐I. It is almost exclusively the result of a GH‐secreting pituitary adenoma. In addition to the somatic features, uncontrolled acromegaly is associated with a number of complications and excess mortality. Management is aimed at control of the tumour; normalization of GH and IGF‐I secretion and relief of symptoms. Initial management of GH‐secreting pituitary adenoma is widely accepted as endonasal trans‐sphenoidal surgery, with second‐line therapy where disease is uncontrolled in most cases being somatostatin analogue therapy. With the combination of surgery and somatostatin analogue therapy, control is achieved in around 75% of patients; however, this leaves a significant proportion of patients requiring multimodality therapy to achieve remission. Within the UK, the health system has finite resources, and decisions for management require consideration of efficacy and cost‐effectiveness. To add to the complexity, subtle differences exist in availability of high‐cost medications used in the treatment of patients with acromegaly across the devolved nations of the UK. In this article, we discuss options for the management of persistent acromegaly following initial surgery and somatostatin analogue therapy, and explore earlier use of dopaminergics and conservative management.

## Graphical abstract

This review covers differences in drug availability across the devolved nations of the UK, the importance and requirement for multimodality therapy to induce remission in around 25% of patients, as well as considering conservative management and more extensive use of cabergoline.

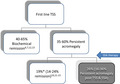

## 1 INTRODUCTION

Acromegaly is the clinical consequence of chronic exposure of the tissues to excessive growth hormone (GH) and IGF‐I. The most frequently reported symptoms of acromegaly include enlargement of the hands and feet, changes in facial appearance, headaches, lethargy, hyperhidrosis, paraesthesia, sexual dysfunction and visual disturbance.^1^ In addition to the classical somatic features, acromegaly is complicated by an increased risk of diabetes mellitus, hypertension, arthropathy, osteoporosis, vertebral fractures, obstructive sleep apnoea, cardiomyopathy and colonic polyps.^2^ Importantly, acromegaly is associated with an increased mortality rate relating predominantly to an excess of respiratory and cardiovascular disease.^3^, ^4^ Acromegaly is almost exclusively the result of a benign pituitary adenoma.

Management is aimed at (a) reducing, or at a minimum stabilizing, the tumour bulk while preserving pituitary hormone function; (b) controlling the excess GH and IGF‐I secretion to obtain a GH level < 1.0 μg/L and normal age‐related IGF‐I level, which has been shown to restore mortality to that expected of the general population^5^; (c) improving the patients' signs and symptoms; and (d) preventing occurrence and/or progression of systemic complications related to long‐term GH excess.^6^, ^7^ Most treatment modalities employed address several of these individual goals. Control of biochemical markers of disease activity, GH and IGF‐I, leads to reduction in mortality, symptoms and many of the recognized complications. Assessment of disease activity is undertaken by measurement of random morning GH and IGF‐I levels; GH nadir during an oral glucose tolerance test (OGTT); or mean GH level during a day curve. The latter is generally carried out by measuring GH levels every 30 minutes over a period of two or three hours. Within the UK, the OGTT is primarily used for the diagnosis of acromegaly and not disease monitoring. Measurement of random morning IGF‐I and GH levels, and day curve mean GH are used to monitor disease activity long‐term.

Within the United Kingdom, health services are provided to the vast majority of the population through the National Health Service (NHS). This is a health service system with finite financial resources. Decisions therefore have to be made regarding allocation of resources and cost‐effectiveness of therapies, particularly concerning high‐cost interventions. The models for assessment of efficacy and cost‐effectiveness of new technologies/medications differ between England and the devolved nations (Scotland, Wales & Northern Ireland). In England, a robust system is underpinned by the National Institute for Health and Care Excellence (NICE), with a further layer of guidance through NHS England specialist commissioning. Assessments in Scotland are undertaken through the Scottish Medicines Consortium (SMC), with Wales (All Wales Medicines Strategy Group; AWMSG) and Northern Ireland (Northern Ireland Formulary; NIF) frequently deferring to guidance given by the SMC. As a consequence, management of disease within the NHS as a whole is likely to have differences in management pathways across nations comprising the UK and when compared with other countries.

Across the UK, it is almost universally accepted that the most efficacious and cost‐effective initial (primary) therapy for acromegaly is pituitary surgery via the trans‐sphenoidal route (TSS) with the aim of inducing remission. Postoperative remission rates for microadenoma in specialist centres approach 90%,^7^ however, are significantly less in macroadenoma,^7^ particularly when there is notable invasion of the cavernous sinus.^7^, ^8^ In these latter cases, remission rates even in specialist centres are <50%.^8^ Most somatotroph adenomas are macroadenomas, and therefore, overall remission rates postoperatively are reported to be 40%‐65%.^6^, ^7^, ^9^, ^10^ The experience of the pituitary surgeon cannot be over emphasized and is a major determinant of achieving biochemical remission.^11^ Reconfiguration of the specialist pituitary services within Manchester, UK, in 2005 led to centralization and reduction in the number of pituitary surgeons. This led to improvements in rates of postoperative biochemical remission from 27% prior to the reconfiguration to 67% following implementation of the changes.^12^, ^13^

A smaller proportion of patients are subject to trans‐sphenoidal surgery (TSS) to debulk the tumour without the prospect of obtaining remission. In these cases, the aim of surgery is to relieve pressure of the tumour on the optic chiasm and other neighbouring structures, or alternatively to reduce the ambient GH and IGF‐I levels to improve efficacy of future nonsurgical management. In the remaining patients where the majority of the tumour is located within the cavernous sinus, or surgery is contraindicated as a consequence of co‐morbidities, consideration is given to alternative therapies. In this scenario, primary medical therapy is usually considered preferable, most commonly utilizing long‐acting somatostatin analogues. Primary radiotherapy (stereotactic or fractionated) can be considered as an alternative treatment, though is used infrequently in this situation.

## 2 PERSISTENT ACROMEGALY

### 2.1 Initial management PostOperatively (second line)

Patients considered as having ‘persistent disease’ comprise those who continue to have GH and/or IGF‐I levels above target either (a) following failed initial surgery directed at inducing remission or (b) those who have undergone debulking surgery to reduce GH and IGF‐I levels to increase the efficacy of future medical therapy. From the aspect of management, those patients who were felt inappropriate for first‐line surgery can also be considered within this cohort of patients.

Options for second‐line treatment of patients who have not achieved remission include repeat surgery, medical therapy or radiotherapy. Within specialist neurosurgical centres with high rates of remission following initial surgery of microadenoma and intrasellar macroadenoma, persistent disease activity is the hallmark of macroadenoma with cavernous sinus invasion.^8^ Intuitively, it would be expected that this would limit the value of further surgical intervention. However, in two retrospective studies examining outcomes of a total of 67 patients who underwent repeat surgery for persistent disease, rates of remission of 57.1% and 58.5% have been reported.^14^, ^15^ Cavernous sinus involvement and tumour segmentation were negative predictors of remission following second trans‐sphenoidal surgery.^15^ These data would support consideration of repeat surgery where the residuum following primary surgery remains intrasellar.^7^

In the majority of patients, however, medical therapy is considered second line. The Endocrine Society guideline recommends the use of first‐generation somatostatin analogues in the majority of patients, cabergoline in those with minimal disease activity and consideration of the use of pegvisomant.^7^ Long‐acting first‐generation somatostatin analogue therapy is, therefore, generally considered the preferred option for individuals with persistent disease post‐TSS. There are currently three somatostatin analogues (SSA) licensed for use in acromegaly; lanreotide, octreotide and pasireotide. Lanreotide and octreotide, together, are considered as first‐generation SSAs, whereas pasireotide is a second‐generation SSA.

Physiological control of GH secretion results from the complex interaction of two hypothalamic hormones: growth hormone‐releasing hormone (GHRH) which is stimulatory and somatostatin which is inhibitory. Somatostatin exerts its biologic effects through five specific membrane‐bound high‐affinity receptor subtypes (SSTR1‐5),^16^ activating intracellular signalling primarily through an inhibitory Gα subunit. GH secretion is predominantly regulated through SSTR2 and SSTR5.^17^ Although somatostatin has been shown to inhibit GH release from GH‐secreting adenoma,^18^ clinical usefulness is limited due to a half‐life of 2‐3 minutes. Two somatostatin analogues, octreotide and lanreotide, have been developed to overcome this limitation.

Considerable long‐term clinical experience has been obtained with the long‐acting formulations of lanreotide and octreotide; lanreotide autogel (ATG) and octreotide LAR, respectively. Availability and use of these analogues is accepted widely across the UK. These two analogues act via high‐affinity binding to SSTR2, with lower affinity binding to SSTR5. Clinically, lanreotide ATG and octreotide LAR have been shown to have similar efficacy,^19^ and outcomes are not dissimilar whether used as primary therapy or following surgery.^20^ Effects on tumour growth are remarkable, with <2% of adenoma showing significant growth on treatment, and symptom control being equivalent to that observed with surgery.^20^ Strict control of GH and IGF‐I secretion is, however, less frequently delivered, with 30%‐40% of patients achieving both a GH < 2.5 μg/L and a normal age‐related IGF‐I level.^19^, ^21^, ^22^, ^23^ Higher baseline GH and IGF‐I levels are associated with a lower proportion of patients achieving target values.^24^ The PRIMARYs study exemplifies the above outcomes.^22^ Ninety patients with acromegaly resulting from a macroadenoma were treated with lanreotide ATG 120 mg every 4 weeks for 48 weeks.^22^ Tumour shrinkage of >20% was achieved in 63%, and although 10% showed some degree of tumour enlargement, only 2% showed enlargement of >20%. The combined end‐point of GH < 2.5 μg/L and normal age‐related IGF‐I was achieved in only 34% of patients.^22^

The most frequent side effects of somatostatin analogues relate to effects on SSTRs within the gastrointestinal (GI) system, namely abdominal cramps and loose stools. Tachyphylaxis to these side effects occurs with repeated injections. Hyper‐ and hypoglycaemia can occur, and long‐term usage is associated with a 15%‐20% rate of cholelithiasis.

### 2.2 Further therapy (third line)

**FIGURE 1:**
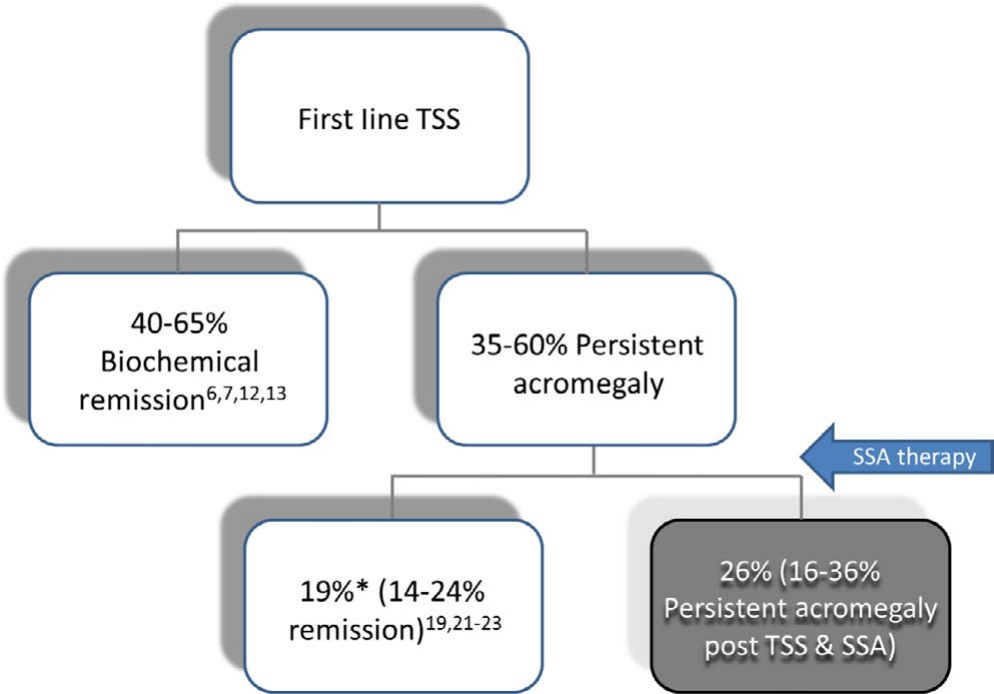
Percentage of the initial cohort of patients with remission and persistent disease following first‐line treatment of acromegaly with trans‐sphenoidal surgery (TSS); and second‐line therapy with somatostatin analogue (SSA) therapy. *Assuming efficacy of SSA therapy of 40% in achieving target GH < 2.5 mg/L and normal age‐related IGF‐I

From the overall cohort of patients presenting with acromegaly, remission would be expected to occur in 40%‐65% following first‐line TSS.^6^, ^7^, ^9^, ^10^ In the 35%‐60% who have evidence of persistent biochemical disease, implementation of SSA therapy would be expected to induce biochemical remission in 30%‐40% of these individuals.^19^, ^21^ Assuming at best that 40% of the 35%‐60% with persistent disease achieve remission with SSA therapy, this would be equivalent to ~19% (14%‐24%) of the original cohort. The combination of first‐line surgery, and second‐line SSA therapy for ‘persistent disease’, is therefore likely to lead to remission in, at most, 64%‐84% of patients overall (Figure 1). These data confirm that a significant proportion of patients with acromegaly, approximating to one in four (26%) will require multimodality therapy to induce remission.

For patients who continue to have active disease despite TSS surgery and maximal dose SSA, there is no preferred option for management. The Endocrine Society guideline suggests consideration of combined drug therapy where there is a partial clinical response to the first drug initiated (usually SSA)^7^ or alternative monotherapy where there has been no clinical or biochemical response to the initial drug used.^7^ In reality, options for further management include (a) SSA dose escalation; (b) addition of cabergoline to the SSA; (c) use of pegvisomant as monotherapy or in combination; (d) pasireotide; and (e) radiotherapy (stereotactic or fractionated). Combinations of these additional therapies are likely to be required in a number of cases.

#### 2.2.1 High dose SSA therapy

Escalation of the dose or dosing frequency of first‐generation SSA can lead to control of GH and IGF‐I in a subset of partial responders to SSA. In a multicentre study, 30 patients who failed to achieve GH < 1.0 μg/L and/or age‐matched IGF‐I < 1.2‐fold the upper limit of normal (ULN) following 6 months lanreotide ATG 120 mg or octreotide LAR 30 mg every 4 weeks were randomized to lanreotide ATG 120 mg every 3 weeks or 180 mg administered every 4 weeks for a total of 24 weeks.^25^ IGF‐I levels of <1.2 ULN were achieved in 27.6%; however, GH values for the cohort remained unchanged and only 3/30 (10%) achieved both biochemical targets.^25^ Adverse events not present at baseline occurred in 63.3% and related to gastrointestinal disturbance and abnormalities of carbohydrate handling. Within the UK, neither lanreotide ATG nor octreotide LAR are however approved or funded for use above the licensed dose or frequency.

#### 2.2.2 Addition of cabergoline to SSA therapy

In healthy men, dopamine, either as a continuous infusion or when infused concurrently with GHRH, significantly increases GH secretion supporting a role of dopamine in the physiological regulation of GH.^26^ In contrast, dopamine is a negative physiological regulator of prolactin secretion. Somatotroph and lactrotroph cells are of common embryological origin, with transcription factors POU1F1 and PROP1 being obligatory to development of both cell lines. Dopaminergic analogues are highly effective in inhibiting prolactin secretion and causes regression of prolactinoma through activation of the dopamine subtype‐2 receptor (D2R). GH‐secreting and mixed mammosomatotroph tumours also express the D2R, though which both prolactin and GH can be regulated.

Following the unexpected observation that administration of L‐dopa in patients with acromegaly led to a reduction in the GH levels, dopamine agonists became the first medical treatment available for acromegaly.^27^ Cabergoline is a dopamine D2 receptor agonist with long duration of action, which allows its administration once or twice weekly in most cases. Studies of the efficacy of the addition of cabergoline to somatostatin analogue therapy have included small numbers of patients and have been of variable design. A meta‐analysis of five studies, three prospective and two retrospective, comprises the most reliable data to date.^28^ Together, these five studies, however, included only 77 patients. Inclusion criteria were failure of SSA to normalize IGF‐I. Baseline GH and IGF‐I levels were 7.4 ± 12.5 μg/L and 70.9 ± 36.6 nmol/L, respectively. SSA therapy had been used after surgery in 74% and as first‐line therapy in 26% of the cohort. The mean cabergoline dose was 2.5 mg/week. Outcome data revealed that the addition of cabergoline normalized age‐adjusted IGF‐I in 52% of the cohort and reduced mean GH levels to 3.6 ± 3.8 μg/L.^28^ The probability of achieving target IGF‐I levels was highest for those with only mild‐moderate disease activity (defined as IGF‐I < 250% of the upper limit of normal); however, significant improvements were observed in IGF‐I even in those with marked elevations of IGF‐I at baseline.

A number of similar studies have been published since the aforementioned meta‐analysis.^29^, ^30^, ^31^, ^32^ In these studies, normalization of IGF‐I levels was achieved in 30%‐48%. Normalization of IGF‐I levels was predicted by lower baseline IGF‐I and GH values, however, was less dependent on co‐secretion of prolactin.

Side effects of dopaminergic agonists relate to activation of dopamine receptors in nonendocrine tissues leading to postural hypotension, nasal congestion, anorexia, nausea and constipation. Cabergoline is an ergot‐derived dopaminergic agonist, and when used in high doses to treat Parkinson's disease (4 mg/day) has been associated with fibrosis, most frequently fibrotic valvular heart disease. In comparison, the doses used in acromegaly are much lower (up to 3.5 mg/week in the majority) and data regarding development of fibrotic valvular heart disease have been reassuring.^33^, ^34^ More recently, the use of dopaminergic agonists has been associated with occurrence of impulse disorders and mood changes.^35^

#### 2.2.3 Pegvisomant therapy

Pegvisomant is a genetically engineered analogue of human growth hormone which acts as a highly selective growth hormone receptor antagonist, blocking GH receptor signalling and subsequent IGF‐I production, thus eliminating the actions of GH on peripheral tissues. The two seminal studies of pegvisomant monotherapy demonstrated efficacy, defined by normalization of age‐related IGF‐I levels, of 89%‐97% of patients.^36^, ^37^ In contrast, real‐world data from the ACROSTUDY showed lower rates of IGF‐I normalization; however, this likely reflects inadequate titration of pegvisomant dosage and lower rates of compliance than seen in the controlled clinical studies.^38^ Adverse events are infrequent, with elevation of liver function transaminases more than three‐fold ULN in 11.3%‐13.5%^39^ being the most frequently reported and necessitating drug withdrawal in a minority of cases.

The first study in 2005 examining the addition of pegvisomant to SSA therapy showed control of IGF‐I in over 95% of individuals.^40^ Similar studies have confirmed these excellent outcomes with almost all patients showing normalization of IGF‐I levels.^41^ As a consequence of continued use of SSA concomitantly with pegvisomant, concerns over enlargement of the pituitary adenoma residuum have not been realized. Whether using a combination of SSAs with pegvisomant allows lower doses of these drugs to be used has been suggested in some, but not all studies.^39^

Pegvisomant has been commissioned by NHS England within the pathway of management of acromegaly who fulfil specific clinical and biochemical criteria.^42^ Patients considered for treatment with pegvisomant are those who have persistent disease, clinically or metabolically, despite pituitary surgery and second‐line SSA therapy. Those patients where surgery is contraindicated or who are unable to tolerate SSA therapy are also considered, irrespective of whether they have had pituitary radiotherapy or not. A further stipulation is that the age‐ and gender‐matched IGF‐I must be greater than 1.3‐fold ULN. The only exclusion criteria are those of life‐threatening complications of acromegaly and an alanine transaminase greater than three‐fold ULN. Discontinuation of pegvisomant is recommended where IGF‐I levels fail to normalize, or at a minimum reduce by 50% after 6 months despite maximum titration. Additionally, drug discontinuation is recommended in patients who are noncompliant, develop drug‐related adverse effects or a severe unrelated life‐threatening condition. In contrast to England, the devolved nations have access to pegvisomant in accord with the licensing agreement. Similarly to England, use of pegvisomant is following unsuccessful surgery and failure to normalize IGF‐I levels with SSA. The main difference to the commissioning in England is that there is no stated requirement for the degree of elevation of the IGF‐I level. Importantly, use in all countries within the UK is contingent on patient access schemes that have been individually agreed with the manufacturer within each of the nations, and that improve cost‐effectiveness.

Pegvisomant monotherapy normalizes IGF‐I in almost all individuals where the dosage is adequately titrated,^36^, ^37^ effectively negating the need for combined therapy to achieve biochemical disease control. Review of the available data relating to dose sparing by concomitant use of SSA as a cost‐effective approach was examined during commissioning of pegvisomant in England. The data were however not felt sufficiently robust to recommend this approach. Use of pegvisomant in combination with SSA falls outside the licensing agreement for pegvisomant and therefore is not approved in Scotland, Wales or Northern Ireland. As such, combined therapy has not been accepted in any of the individual countries of the UK.

#### 2.2.4 Pasireotide

SSTR subtypes form both homo‐ and heterodimers suggesting crosstalk between the receptor subtypes.^43^ Furthermore, combinations of SSTR2 and SSTR5 specific ligands are synergistic for GH inhibition from GH‐secreting adenoma, leading to greater inhibition of GH than either ligand alone or a combination of two ligands specific for the same receptor subtype. SSA also have peripheral effects to reduce GH‐induced IGF‐I production directly at the liver via the SSTR2 and SSTR3 receptor subtypes.^44^ Pasireotide is a second‐generation SSA that displays high‐affinity binding to human SSTR1, 2, 3 and 5, with 30‐40 fold higher affinity for SSTR1 and SSTR5, though slightly lower affinity for SSTR2, compared with octreotide and lanreotide.^45^ Based on these data, the multiligand‐binding pasireotide has been considered a promising candidate to improve control of GH and IGF‐I levels in patients with acromegaly.

The initial phase II clinical study comparing subcutaneous pasireotide and octreotide suggested pasireotide to have the greater efficacy in controlling GH and IGF‐I levels.^46^ The follow‐up phase III study of the long‐acting formulation, pasireotide LAR, showed greater efficacy than octreotide LAR in achieving target GH levels of <2.5 μg/L and normal IGF‐I levels (31.3% vs 19.2%, respectively) in patients with uncontrolled acromegaly who had not previously received medical therapy.^23^ Additionally, pasireotide LAR has been shown to induce biochemical remission in up to 20% of patients who remain uncontrolled during long‐term therapy with first‐generation SSAs.^47^

Adverse events were similar for pasireotide and first‐generation SSA with the exception of hyperglycaemia, which was significantly more frequent with pasireotide, occurring in up to 67% of patients.^23^, ^47^ The development of diabetes and hyperglycaemia with pasireotide is an obvious concern, as both type 1 and type 2 diabetes themselves are associated with an increased standardized mortality ratio (SMR). However, it does appear that the diabetes associated with pasireotide may be reversible with treatment discontinuation, and that mechanistically it differs from other forms of diabetes. Studies in healthy subjects have shown pasireotide to inhibit insulin and incretin secretion, with less pronounced inhibition of glucagon secretion and no change in hepatic or peripheral insulin sensitivity.^48^

Usage of pasireotide in the management of acromegaly was not routinely commissioned by NHS England^49^ based on the balance of benefit and risk, however, has been accepted as a viable third‐line treatment option by the SMC, AWMSG and NIF. The SMC recommends usage in adult patients where surgery is not an option or has been unsuccessful, and who are inadequately controlled by first‐generation SSAs.^50^

#### 2.2.5 Radiotherapy

Conventional fractionated conformal radiotherapy (XRT) delivers charged particles (photons) with high precision to the tumour generally using a linear accelerator. Techniques have significantly evolved as a consequence of improvements in focusing (multileaf collimator), number of beams, immobilization, imaging and planning. Use in the management of acromegaly has shown XRT to be highly effective in preventing growth of somatotroph tumours in 80%‐90% of individuals with acromegaly at 10 years.^51^ In contrast, control of GH and IGF‐I levels occurs at a more sedate rate with 50%‐60% of individuals achieving remission at 10 years.^51^ In the largest individual study to date, retrospective data from 656 patients with acromegaly showed GH values < 2.5 μg/L and IGF‐I normalization to be achieved in 36% and 50% of patients at 5 years respectively; and 60% and 63% at 10 years, respectively.^52^ Following XRT, mean GH levels decrease by around 50% every 2 years; however, IGF‐I levels decrease at a slower rate.^53^, ^54^ The rate at which target GH levels are achieved is therefore dependent upon the ambient GH level at the time of XRT. Pituitary radiotherapy has been reported to be associated with increased risk for cerebrovascular disease, optic neuritis, visual loss and necrosis of the normal brain tissue in only occasional series and at a very low prevalence; secondary tumours (meningioma and glioma) are reported in 2%‐3% at 10‐20 years; and a variable degree of hypopituitarism in 50%‐60% with long‐term follow‐up.^51^, ^52^, ^53^, ^54^, ^55^, ^56^ An excess mortality has additionally been described in patients with acromegaly who have received XRT.^57^ It remains unclear whether all the described excess mortality relates to XRT or the selection of patients with more aggressive tumours to undergo radiotherapy. With evolution of radiotherapy techniques, it is likely that many of these adverse effects will occur less frequently. Furthermore, use of advanced forms of 3D conformal radiotherapy such as intensity‐modulated radiation therapy (IMRT) achieves a higher degree of target conformity and greater sparing of the surrounding tissues to radiation, and may further reduce the putative adverse effects of XRT.

Stereotactic radiosurgery (SRS) has been introduced with the aim of reducing exposure of the normal tissue surrounding the pituitary adenoma, whilst maintaining the effectiveness. SRS is most frequently delivered from multiple colbalt^58^ gamma‐emitting sources or a modified linear accelerator as a single fraction, adding patient convenience to this technique. Control of tumour growth, GH and IGF‐I levels, as well as adverse sequelae of SRS, do not appear to be markedly divergent from XRT.^51^ Differences in outcomes of studies of SRS and XRT are potentially explicable by patient selection and pretreatment GH and IGF‐I values. SRS is used in smaller tumours, at least 3 mm from critical structures such as the optic chiasm. Studies show a similar rate of fall of GH and IGF‐I levels with both techniques.^59^, ^60^ Particle radiation with proton therapy has been utilized in patients with acromegaly to further improve conformality of dose and reduce radiation exposure of the surrounding tissues.^58^, ^61^ Larger studies and longer duration of follow‐up will however be required to determine if proton therapy is superior to use of photons in the control of somatotroph tumour growth, hormonal secretion and adverse effects.

### 2.3 Alternative management options

#### 2.3.1 Cabergoline monotherapy

Dopaminergic drugs when used as monotherapy in patients with somatotroph tumours paradoxically show suppression of GH secretion.^26^, ^27^ As a consequence, dopaminergic drugs have been trialled in the management of acromegaly, not only as combined therapy with SSA (as discussed), but also as monotherapy. The first dopaminergic drug to be used in management of acromegaly was bromocriptine. Studies in small numbers of patients showed very limited efficacy with only around 10% achieving biochemical target. With the advent of cabergoline and quinagolide for treatment of prolactinomas came increased efficacy, fewer side effects and a more acceptable dosing regimen. Early studies of the use of cabergoline in patients with acromegaly included small numbers of patients and showed variable efficacy, with normalization of GH levels in 0%‐100%. Inadequate studies, combined with the historically poor efficacy of bromocriptine in individuals with acromegaly, have led to this class of drugs being considered only infrequently as monotherapy for controlling GH and IGF‐I levels. Their use now generally being accepted for patients with only mild disease activity, or as an addition to SSA therapy.

A meta‐analysis of 10 studies (n = 160) in 2011 reviewed the published data regarding the effects of cabergoline monotherapy in the management of acromegaly. All but one of the studies were open, and none were randomized or placebo‐controlled. Cabergoline was used first line in 21% of patients, and the overall mean dose was 2.6 mg/week. Baseline GH and IGF‐I levels were 16 ± 34 μg/L and IGF‐I 82.9 ± 36.5 nmol/L, respectively. Target GH levels of <2.5 μg/L and a normal age‐adjusted IGF‐I were achieved in 48% and 34%, respectively.^28^ Patients who achieved target GH and IGF‐I levels had lower baseline IGF‐I levels and elevated prolactin levels at baseline. Data from the UK Acromegaly Register examined 355 courses of treatment with cabergoline, and of these, 36% achieved a target GH < 2.0 μg/L and a normal IGF‐I level.^24^

#### 2.3.2 Conservative management

Consideration needs to be given to how rigidly target GH and IGF‐I values are strived for. Although achieving GH/IGF‐I control is critical for optimal outcomes, other factors that need to be considered are patient's quality of life, potential side effects or complications of therapeutic interventions, as well as the cost‐effectiveness of treatment. For example, in an individual with a normal age‐adjusted IGF‐I but GH value of 1.6 μg/L, is there a need for further targeted therapy to reduce the GH levels further? An alternative, in the absence of symptoms, may be to simply monitor these biological markers whilst undertaking surveillance for complications (obstructive sleep apnoea, cardiomyopathy), and aggressively managing any risk factors (hypertension, diabetes, lipid anomalies, etc). This course of action may be more relevant where acromegaly is diagnosed late in life. To date, no studies have been directed at this course of management.

### 2.4 Cost considerations

In a health system with finite resources, it is important that efficacy and impact on mortality and morbidity are considered alongside cost of the intervention when deciding on the most appropriate therapy for an individual. Control of symptoms, tumour growth and long‐term morbidity and mortality, however, take precedent in any clinical decision‐making. The most cost‐effective treatment remains TSS when remission is achieved. This emphasizes the importance of surgery being undertaken by specialist pituitary surgeons who achieve the highest rates of remission. Life‐long medical therapy for persistent disease activity following TSS can lead to significant associated costs, particularly for younger patients. The long‐term cost of medical therapy can potentially be offset by radiation therapy which would limit the duration that medical therapy is required. Significant concerns over the development of premature cerebrovascular disease have led to a decline in use of radiation therapy; however, stereotactic radiotherapy by utilizing multiple beams of low‐dose radiotherapy will limit the radiation dose to individual areas of normal brain tissue, and potentially mitigate the risk of cerebrovascular disease. Stereotactic radiotherapy should therefore be considered in all patients who continue to have active disease after TSS.

Control of GH and IGF‐I levels with cabergoline is observed in a not dissimilar proportion of patients to those receiving somatostatin analogue therapy. It may therefore be appropriate that cabergoline should be trialled before SSA therapy in disease management based on efficacy and cost‐effectiveness. Current NHS costs for 28 days therapy with octreotide LAR or lanreotide ATG approximate to £500‐£1000. In contrast, cabergoline at a weekly dose of 2‐3 mg equates to £25‐£40 for the same period (Table 1).

**TABLE 1:** ‘NHS indicative price’ of medications used in management of acromegaly

	Dosage	Cost for 28 d
Cabergoline (mg/wk)	0.5 mg weekly	£17.50
	1.0 (0.5 mg × 2/wk)	£35.00
	2.0 (1.0 mg × 2/wk)	£33.20
	3.5 (0.5 mg daily)	£122.50
Octreotide LAR (mg/28 d)	10 mg	£549.71
	20 mg	£799.33
	30 mg	£998.41
Lanreotide ATG (mg/28 d)	60 mg	£551.00
	90 mg	£736.00
	120 mg	£937.00
Pasireotide LAR (mg/28 d)	40 mg	£2300.00
	60 mg	£2300.00
Pegvisomant (mg/day)	10 mg	£1400.00
	20 mg	£2800.00
	30 mg	£4200.00

Note

Costs taken from British National Formulary (BNF) on‐line, but do not take account of patient access schemes.

Recent commissioning for pegvisomant in the devolved nations aimed at establishing the position within treatment algorithms and has involved concurrent discussions with the manufacturer, Pfizer Inc, to establish patient access schemes within the NHS. These access schemes have led to negotiated discounts in cost of pegvisomant to enable cost‐effective usage.

## 3 CONCLUSIONS

From these data, it is important to note that many individuals with acromegaly will require multimodality therapy to obtain remission. Primary therapy remains surgery in the majority of cases, followed by somatostatin analogue therapy where remission is not achieved. Thereafter, approximately one in four patients will require at least one further intervention. Therapeutic options include repeat surgery, medical therapy and radiation, with combinations of these modalities and medical therapies often being necessary. There are subtle differences in availability of high‐cost medical therapies (pegvisomant and pasireotide) between the devolved nations, with greatest limitations to use within England. These differences are, however, unlikely to affect management of the vast majority of patients in whom pegvisomant or pasireotide are not required to achieve symptom, tumoral and biochemical control.

Analogous to medical treatment of hypertension or diabetes, where basal values are further from target, single agent therapy is less likely to achieve remission whatever class of drug is utilized. Data on the efficacy and cost of cabergoline would suggest a trial of this drug should be considered before SSA therapy, particularly when GH and IGF‐I values are not grossly elevated. In the latter cases, it is likely that combined therapy will be required. More robust studies are however required with randomization against SSA therapy to determine relative efficacy of cabergoline, and thereby solidify the place of cabergoline within the management algorithm for patients with acromegaly.

Potential adverse effects of conformal fractionated radiotherapy, in particular evolving hypopituitarism and the putative association with cerebrovascular disease and increased mortality, have led to a decline in usage of this therapeutic modality. Stereotactic radiosurgery has not been shown to have superiority over conformal fractionated radiotherapy in the rate of achieving tumoral or biochemical control of somatotroph tumours. SRS reduces the exposure of surrounding normal tissue to photons and may potentially reduce adverse effects; however, long‐term data are required to confirm this.

Finally, while recognizing the importance of managing complications and risk factors associated with acromegaly, it may be that aggressive drug therapy in those with GH and/or IGF‐I values only marginally above target may not be warranted, particularly in those who develop disease later in life. Further study of the impact of management directed towards complications and risk factors on long‐term outcomes of patients with acromegaly needs further investigation.

## CONFLICT OF INTEREST

NK, KS, and AH have nothing to disclose. RDM has research funding from Pfizer, Ipsen, and Sandoz. There has been no funding associated with this work.

## AUTHOR CONTRIBUTION

RDM conceived and presented the idea for the review. NK, KS, AH and RDM helped to focus the concept and direction of the review. All authors aided with searches and assessed the relevant literature. NK and RDM wrote the first draft of the manuscript. All authors contributed to review and revision of the initial manuscript and subsequent revisions.

## ETHICAL APPROVAL

There are no ethical concerns as this is a review of published data.

## Data Availability

There are no new data associated with this manuscript.

## References

[1] Molitch ME . Clinical manifestations of acromegaly. Endocrinol Metab Clin North Am. 1992;21(3):597‐614. 1521514

[2] Gadelha MR , Kasuki L , Lim DST , Fleseriu M . Systemic complications of acromegaly and the impact of the current treatment landscape: an update. Endocr Rev. 2019;40(1):268‐332. 30184064 10.1210/er.2018-00115

[3] Holdaway IM , Bolland MJ , Gamble GD . A meta‐analysis of the effect of lowering serum levels of GH and IGF‐I on mortality in acromegaly. Eur J Endocrinol. 2008;159(2):89‐95. 18524797 10.1530/EJE-08-0267

[4] Orme SM , McNally RJ , Cartwright RA , Belchetz PE . Mortality and cancer incidence in acromegaly: a retrospective cohort study. United Kingdom Acromegaly Study Group. J Clin Endocrinol Metab. 1998;83(8):2730‐2734. 9709939 10.1210/jcem.83.8.5007

[5] Holdaway IM , Rajasoorya RC , Gamble GD . Factors influencing mortality in acromegaly. J Clin Endocrinol Metab. 2004;89(2):667‐674. 14764779 10.1210/jc.2003-031199

[6] Melmed S , Bronstein MD , Chanson P , et al. A Consensus Statement on acromegaly therapeutic outcomes. Nat Rev Endocrinol. 2018;14(9):552‐561. 30050156 10.1038/s41574-018-0058-5 PMC7136157

[7] Katznelson L , Laws ER Jr , Melmed S , et al. Acromegaly: an endocrine society clinical practice guideline. J Clin Endocrinol Metab. 2014;99(11):3933‐3951. 25356808 10.1210/jc.2014-2700

[8] Briceno V , Zaidi HA , Doucette JA , et al. Efficacy of transsphenoidal surgery in achieving biochemical cure of growth hormone‐secreting pituitary adenomas among patients with cavernous sinus invasion: a systematic review and meta‐analysis. Neurol Res. 2017;39(5):387‐398. 28301972 10.1080/01616412.2017.1296653

[9] Minniti G , Jaffrain‐Rea ML , Esposito V , Santoro A , Tamburrano G , Cantore G . Evolving criteria for post‐operative biochemical remission of acromegaly: can we achieve a definitive cure? An audit of surgical results on a large series and a review of the literature. Endocr Relat Cancer. 2003;10(4):611‐619. 14713271 10.1677/erc.0.0100611

[10] Chen CJ , Ironside N , Pomeraniec IJ , et al. Microsurgical versus endoscopic transsphenoidal resection for acromegaly: a systematic review of outcomes and complications. Acta Neurochir (Wien). 2017;159(11):2193‐2207. 28913667 10.1007/s00701-017-3318-6 PMC6558977

[11] Wass JA , Turner HE , Adams CB . The importance of locating a good pituitary surgeon. Pituitary. 1999;2(1):51‐54. 11081172 10.1023/a:1009982232672

[12] Lissett CA , Peacey SR , Laing I , Tetlow L , Davis JR , Shalet SM . The outcome of surgery for acromegaly: the need for a specialist pituitary surgeon for all types of growth hormone (GH) secreting adenoma. Clin Endocrinol (Oxford). 1998;49(5):653‐657. 10.1046/j.1365-2265.1998.00581.x 10197082

[13] Wang YY , Higham C , Kearney T , Davis JR , Trainer P , Gnanalingham KK . Acromegaly surgery in Manchester revisited‐the impact of reducing surgeon numbers and the 2010 consensus guidelines for disease remission. Clin Endocrinol (Oxford). 2012;76(3):399‐406. 10.1111/j.1365-2265.2011.04193.x 21824170

[14] Wilson TJ , McKean EL , Barkan AL , Chandler WF , Sullivan SE . Repeat endoscopic transsphenoidal surgery for acromegaly: remission and complications. Pituitary. 2013;16(4):459‐464. 23307479 10.1007/s11102-012-0457-x

[15] Yamada S , Fukuhara N , Oyama K , Takeshita A , Takeuchi Y . Repeat transsphenoidal surgery for the treatment of remaining or recurring pituitary tumors in acromegaly. Neurosurgery. 2010;67(4):949‐956. 20881560 10.1227/NEU.0b013e3181ec4379

[16] Ben‐Shlomo A , Melmed S . Pituitary somatostatin receptor signaling. Trends Endocrinol Metab. 2010;21(3):123‐133. 20149677 10.1016/j.tem.2009.12.003 PMC2834886

[17] Miller GM , Alexander JM , Bikkal HA , Katznelson L , Zervas NT , Klibanski A . Somatostatin receptor subtype gene expression in pituitary adenomas. J Clin Endocrinol Metab. 1995;80(4):1386‐1392. 7714115 10.1210/jcem.80.4.7714115

[18] Yen SS , Siler TM , DeVane GW . Effect of somatostatin in patients with acromegaly: suppression of growth hormone, prolactin, insulin and glucose levels. N Engl J Med. 1974;290(17):935‐938. 4593614 10.1056/NEJM197404252901704

[19] Murray RD , Melmed S . A critical analysis of clinically available somatostatin analog formulations for therapy of acromegaly. J Clin Endocrinol Metab. 2008;93(8):2957‐2968. 18477663 10.1210/jc.2008-0027

[20] Colao A , Cappabianca P , Caron P , et al. Octreotide LAR vs. surgery in newly diagnosed patients with acromegaly: a randomized, open‐label, multicentre study. Clin Endocrinol (Oxford). 2009;70(5):757‐768. 10.1111/j.1365-2265.2008.03441.x 19178516

[21] Kyriakakis N , Chau V , Lynch J , Orme SM , Murray RD . Lanreotide autogel in acromegaly ‐ a decade on. Expert Opin Pharmacother. 2014;15(18):2681‐2692. 25307803 10.1517/14656566.2014.970173

[22] Caron PJ , Bevan JS , Petersenn S , et al. Tumor shrinkage with lanreotide Autogel 120 mg as primary therapy in acromegaly: results of a prospective multicenter clinical trial. J Clin Endocrinol Metab. 2014;99(4):1282‐1290. 24423301 10.1210/jc.2013-3318 PMC4009579

[23] Colao A , Bronstein MD , Freda P , et al. Pasireotide versus octreotide in acromegaly: a head‐to‐head superiority study. J Clin Endocrinol Metab. 2014;99(3):791‐799. 24423324 10.1210/jc.2013-2480 PMC3965714

[24] Howlett TA , Willis D , Walker G , Wass JA , Trainer PJ . Control of growth hormone and IGF1 in patients with acromegaly in the UK: responses to medical treatment with somatostatin analogues and dopamine agonists. Clin Endocrinol (Oxford). 2013;79(5):689‐699. 10.1111/cen.12207 23574573

[25] Giustina A , Mazziotti G , Cannavo S , et al. High‐dose and high‐frequency lanreotide autogel in acromegaly: a randomized, multicenter study. J Clin Endocrinol Metab. 2017;102(7):2454‐2464. 28419317 10.1210/jc.2017-00142

[26] Vance ML , Kaiser DL , Frohman LA , Rivier J , Vale WW , Thorner MO . Role of dopamine in the regulation of growth hormone secretion: dopamine and bromocriptine augment growth hormone (GH)‐releasing hormone‐stimulated GH secretion in normal man. J Clin Endocrinol Metab. 1987;64(6):1136‐1141. 3553220 10.1210/jcem-64-6-1136

[27] Liuzzi A , Chiodini PG , Botalla L , Cremascoli G , Silvestrini F . Inhibitory effect of L‐Dopa on GH release in acromegalic patients. J Clin Endocrinol Metab. 1972;35(6):941‐943. 4634493 10.1210/jcem-35-6-941

[28] Sandret L , Maison P , Chanson P . Place of cabergoline in acromegaly: a meta‐analysis. J Clin Endocrinol Metab. 2011;96(5):1327‐1335. 21325455 10.1210/jc.2010-2443

[29] Mattar P , Alves Martins MR , Abucham J . Short‐ and long‐term efficacy of combined cabergoline and octreotide treatment in controlling IGF‐I levels in acromegaly. Neuroendocrinology. 2010;92(2):120‐127. 10.1159/000317314 20802256

[30] Vilar L , Azevedo MF , Naves LA , et al. Role of the addition of cabergoline to the management of acromegalic patients resistant to longterm treatment with octreotide LAR. Pituitary. 2011;14(2):148‐156. 21104199 10.1007/s11102-010-0272-1

[31] Suda K , Inoshita N , Iguchi G , et al. Efficacy of combined octreotide and cabergoline treatment in patients with acromegaly: a retrospective clinical study and review of the literature. Endocr J. 2013;60(4):507‐515. 23291436

[32] Puig‐Domingo M , Soto A , Venegas E , et al. Use of lanreotide in combination with cabergoline or pegvisomant in patients with acromegaly in the clinical practice: the ACROCOMB study. Endocrinol Nutr. 2016;63(8):397‐408. 27448708 10.1016/j.endonu.2016.05.010

[33] Drake WM , Stiles CE , Howlett TA , Toogood AA , Bevan JS , Steeds RP . A cross‐sectional study of the prevalence of cardiac valvular abnormalities in hyperprolactinemic patients treated with ergot‐derived dopamine agonists. J Clin Endocrinol Metab. 2014;99(1):90‐96. 24187407 10.1210/jc.2013-2254 PMC5137780

[34] Drake WM , Stiles CE , Bevan JS , et al. A follow‐up study of the prevalence of valvular heart abnormalities in hyperprolactinemic patients treated with cabergoline. J Clin Endocrinol Metab. 2016;101(11):4189‐4194. 27571182 10.1210/jc.2016-2224

[35] Ioachimescu AG , Fleseriu M , Hoffman AR , Vaughan Iii TB , Katznelson L . Psychological effects of dopamine agonist treatment in patients with hyperprolactinemia and prolactin‐secreting adenomas. Eur J Endocrinol. 2019;180(1):31‐40. 30400048 10.1530/EJE-18-0682

[36] van der Lely AJ , Hutson RK , Trainer PJ , et al. Long‐term treatment of acromegaly with pegvisomant, a growth hormone receptor antagonist. Lancet. 2001;358(9295):1754‐1759. 11734231 10.1016/s0140-6736(01)06844-1

[37] Trainer PJ , Drake WM , Katznelson L , et al. Treatment of acromegaly with the growth hormone‐receptor antagonist pegvisomant. N Engl J Med. 2000;342(16):1171‐1177. 10770982 10.1056/NEJM200004203421604

[38] van der Lely AJ , Biller BM , Brue T , et al. Long‐term safety of pegvisomant in patients with acromegaly: comprehensive review of 1288 subjects in ACROSTUDY. J Clin Endocrinol Metab. 2012;97(5):1589‐1597. 22362824 10.1210/jc.2011-2508

[39] Neggers SJ , Franck SE , de Rooij FW , et al. Long‐term efficacy and safety of pegvisomant in combination with long‐acting somatostatin analogs in acromegaly. J Clin Endocrinol Metab. 2014;99(10):3644‐3652. 24937542 10.1210/jc.2014-2032

[40] Feenstra J , de Herder WW , ten Have SM , et al. Combined therapy with somatostatin analogues and weekly pegvisomant in active acromegaly. Lancet. 2005;365(9471):1644‐1646. 15885297 10.1016/S0140-6736(05)63011-5

[41] Franck SE , Muhammad A , van der Lely AJ , Neggers SJ . Combined treatment of somatostatin analogues with pegvisomant in acromegaly. Endocrine. 2016;52(2):206‐213. 26661938 10.1007/s12020-015-0810-8 PMC4824818

[42] NHSE . Commissioning Policy: Pegvisomant for acromegaly as a third‐line treatment (adults). In Commissioning NS, ed. NHS England; 2016 https://www.england.nhs.uk/wp‐content/uploads/2016/12/clin‐comm‐pol‐16050P.pdf. Accessed December 12, 2016

[43] Rocheville M , Lange DC , Kumar U , Sasi R , Patel RC , Patel YC . Subtypes of the somatostatin receptor assemble as functional homo‐ and heterodimers. J Biol Chem. 2000;275(11):7862‐7869. 10713101 10.1074/jbc.275.11.7862

[44] Murray RD , Kim K , Ren SG , Chelly M , Umehara Y , Melmed S . Central and peripheral actions of somatostatin on the growth hormone‐IGF‐I axis. J Clin Invest. 2004;114(3):349‐356. 15286801 10.1172/JCI19933 PMC484973

[45] Bruns C , Lewis I , Briner U , Meno‐Tetang G , Weckbecker G . SOM230: a novel somatostatin peptidomimetic with broad somatotropin release inhibiting factor (SRIF) receptor binding and a unique antisecretory profile. Eur J Endocrinol. 2002;146(5):707‐716. 11980628 10.1530/eje.0.1460707

[46] Petersenn S , Schopohl J , Barkan A , et al. Pasireotide (SOM230) demonstrates efficacy and safety in patients with acromegaly: a randomized, multicenter, phase II trial. J Clin Endocrinol Metab. 2010;95(6):2781‐2789. 20410233 10.1210/jc.2009-2272

[47] Gadelha MR , Bronstein MD , Brue T , et al. Pasireotide versus continued treatment with octreotide or lanreotide in patients with inadequately controlled acromegaly (PAOLA): a randomised, phase 3 trial. Lancet Diabetes Endocrinol. 2014;2(11):875‐884. 25260838 10.1016/S2213-8587(14)70169-X

[48] Henry RR , Ciaraldi TP , Armstrong D , Burke P , Ligueros‐Saylan M , Mudaliar S . Hyperglycemia associated with pasireotide: results from a mechanistic study in healthy volunteers. J Clin Endocrinol Metab. 2013;98(8):3446‐3453. 23733372 10.1210/jc.2013-1771

[49] NHSE . Clinical Commissioning Policy: Pasireotide for acromegaly as third‐line treatment (adults). In: Commissioning NS, ed. NHS England; 2016 https://www.england.nhs.uk/wp‐content/uploads/2018/07/Pasireotide‐for‐acromegaly‐as‐third‐line‐treatments‐adults.pdf. Accessed July 26, 2016

[50] SMC . Pasireotide (as pamoate), 20 mg, 40 mg 60 mg powder and solvent for suspension for injection (Signifor^®^). SMC No. (1048/15). 2015 https://www.scottishmedicines.org.uk/media/2119/pasireotide__signifor__final_may_2015_amend_020915_for_website.pdf

[51] Minniti G , Scaringi C , Enrici RM . Radiation techniques for acromegaly. Radiat Oncol. 2011;6:167. 22136376 10.1186/1748-717X-6-167 PMC3275813

[52] Jenkins PJ , Bates P , Carson MN , Stewart PM , Wass JA . Conventional pituitary irradiation is effective in lowering serum growth hormone and insulin‐like growth factor‐I in patients with acromegaly. J Clin Endocrinol Metab. 2006;91(4):1239‐1245. 16403824 10.1210/jc.2005-1616

[53] Barrande G , Pittino‐Lungo M , Coste J , et al. Hormonal and metabolic effects of radiotherapy in acromegaly: long‐term results in 128 patients followed in a single center. J Clin Endocrinol Metab. 2000;85(10):3779‐3785. 11061538 10.1210/jcem.85.10.6870

[54] Biermasz NR , van Dulken H , Roelfsema F . Long‐term follow‐up results of postoperative radiotherapy in 36 patients with acromegaly. J Clin Endocrinol Metab. 2000;85(7):2476‐2482. 10902796 10.1210/jcem.85.7.6699

[55] Minniti G , Traish D , Ashley S , Gonsalves A , Brada M . Risk of second brain tumor after conservative surgery and radiotherapy for pituitary adenoma: update after an additional 10 years. J Clin Endocrinol Metab. 2005;90(2):800‐804. 15562021 10.1210/jc.2004-1152

[56] Ayuk J , Clayton RN , Holder G , Sheppard MC , Stewart PM , Bates AS . Growth hormone and pituitary radiotherapy, but not serum insulin‐like growth factor‐I concentrations, predict excess mortality in patients with acromegaly. J Clin Endocrinol Metab. 2004;89(4):1613‐1617. 15070920 10.1210/jc.2003-031584

[57] Sherlock M , Reulen RC , Alonso AA , et al. ACTH deficiency, higher doses of hydrocortisone replacement, and radiotherapy are independent predictors of mortality in patients with acromegaly. J Clin Endocrinol Metab. 2009;94(11):4216‐4223. 19808848 10.1210/jc.2009-1097

[58] Petit JH , Biller BM , Coen JJ , et al. Proton stereotactic radiosurgery in management of persistent acromegaly. Endocr Pract. 2007;13(7):726‐734. 18194929 10.4158/EP.13.7.726

[59] Attanasio R , Epaminonda P , Motti E , et al. Gamma‐knife radiosurgery in acromegaly: a 4‐year follow‐up study. J Clin Endocrinol Metab. 2003;88(7):3105‐3112. 12843150 10.1210/jc.2002-021663

[60] Castinetti F , Taieb D , Kuhn JM , et al. Outcome of gamma knife radiosurgery in 82 patients with acromegaly: correlation with initial hypersecretion. J Clin Endocrinol Metab. 2005;90(8):4483‐4488. 15899958 10.1210/jc.2005-0311

[61] Wattson DA , Tanguturi SK , Spiegel DY , et al. Outcomes of proton therapy for patients with functional pituitary adenomas. Int J Radiat Oncol Biol Phys. 2014;90(3):532‐539. 25194666 10.1016/j.ijrobp.2014.06.068

